# A plasmonic thermal sensing based portable device for lateral flow assay detection and quantification

**DOI:** 10.1186/s11671-019-3240-3

**Published:** 2020-01-13

**Authors:** Zhuo Qu, Kan Wang, Gabriel Alfranca, Jesús M. de la Fuente, Daxiang Cui

**Affiliations:** 1 Institute of Nano Biomedicine and Engineering, Shanghai Engineering Research Center for Intelligent diagnosis and treatment instrument, Department of Instrument Science and Engineering, School of Electronic Information and Electrical Engineering, Shanghai Jiao Tong University, 800 Dongchuan Rd, Shanghai, 200240 China; 2 Instituto de Ciencia de Materiales de Aragón (ICMA), CSIC/Universidad de Zaragoza, C/Pedro Cerbuna 12, 50009 Zaragoza, Spain; 3 Centro de Investigación Biomédica en Red de Bioingeniería, Biomateriales Nanomedicina (CIBER-BBN), 50018 Madrid, Spain

**Keywords:** POCT, LFA, Plasmonic thermal sensing, Biomarker quantification

## Abstract

Point-of-care testing (POCT) is widely used for early diagnosis and monitoring of diseases. Lateral flow assay (LFA) is a successfully commercial tool for POCT. However, LFA often suffers from a lack of quantification and analytical sensitivity. To solve these drawbacks, we have previously developed a thermal LFA using plasmonic gold nanoparticles for thermal contrast into a portable device. Although this methodology significantly improves the analytical sensitivity compared with conventional visual detection, quantification problems are still remaining. In this study, we optimized the operating conditions for the device using conduction and radiation thermal sensing modes allowing the quantification of LFA. The limit of detection of the strips merely containing nanoparticles was decreased by 5-fold (conduction mode) and 12-fold (radiation mode) compared to traditional visual detection. The effect of the ambient temperature was studied for both methods of detection showing that the radiation mode was more affected by the ambient temperature than the conduction mode. To validate the thermal sensing method, human chorionic gonadotropin (HCG) biomarker was quantified using our LFA strips, obtaining a detection limit of 2.8 mIU/mL when using the radiation method of detection.

## Introduction

Early detection and rapid diagnosis are important for disease screening and treatment. Most medical tests are time-consuming and require complicated preparation of clinical samples, large instruments, and well-trained laboratory professionals [1]. Those requirements have greatly hindered the medical treatment in resource-limited areas. Point-of-care testing (POCT) utilizes simple equipment and minimizes the time required to obtain clinically relevant results, allowing clinicians and patients to make decisions quickly. POCT has some obvious advantages, such as short detection time, rapid sample processing, simple instrumentation, and low operation requirements [2, 3]. Thus, the emergence of POCT can help early and rapid diagnosis of diseases, particularly in resource-limited areas, thereby improving medical conditions. However, low analytical sensitivity, complicated operation procedures, and high equipment costs usually hamper the application of this technique. Therefore, further work is urgently needed to find POCT applications with most of the ideal characteristics, while minimizing the drawbacks.

**Scheme 1:**
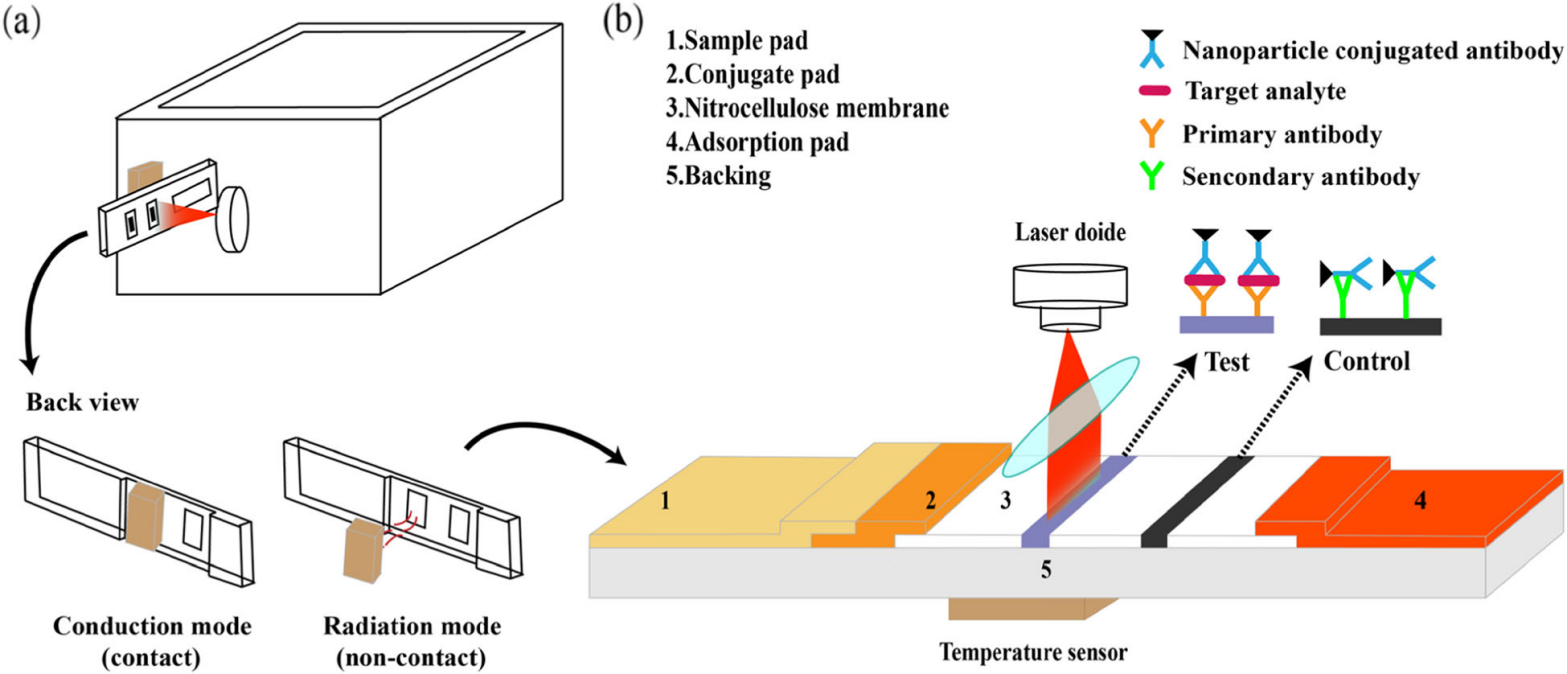
Concept of plasmonic thermal sensing. **a** The model of portable device and the main components (top) with two different sensing modes (bottom). **b** LFA under thermal sensing setting

To solve some of those problems, lateral flow assay (LFA) is a very good candidate as a testing tool in POCT. LFA is a paper-based, point-of-care strip biosensor used to identify target analytes in a given sample [4, 5]. LFA is performed in a paper-based strip (Scheme 1b), which is comprised of a sample pad, conjugate pad, absorption pad, and a nitrocellulose membrane where the detection occurs. Among the advantages of LFA, it is worth mentioning its rapidity and single-step assay, cost-effectiveness, easy operation, small sample volume, and long shelf life under different environmental conditions [6, 7]. Conventional LFA provides “yes or no” results by inspection of color changes on the test line by the naked eye, the most popular method of detection for these kinds of assays. Thus, this type of approach tends to suffer from a lack of accuracy and subjective judgment [8]. Nonetheless, since it is easy to integrate LFA with electronic devices, a feasible detection approach is to develop strip readers in order to obtain accurate quantitative results. Charge-coupled devices (CCD) or complementary metal-oxide semiconductor (CMOS) sensors are usually applied to capture images in strip readers. Image processing software is often adopted to achieve quantitative results. In these optical readers, the optical information obtained from reflection, transmission, or scattering of the light from an external source is recorded to allow for quantification [9–12]. In colorimetric readers, the color intensity, such as grey value or RGB coordinates, is collected from the test and control lines to analyze the LFA strips [13–17]. One drawback of this approach is that the dye may lose its color over time by photodamage, mechanical means, or other degradation processes, resulting in poor repeatability and accuracy. In those systems that resort to fluorescence readers [18, 19], the organic fluorophores are exposed to a specific excitation wavelength that induces the emission of the fluorophore present in the strips at a longer wavelength. This emitted light is then collected to get a quantitative detection. The problem that cannot be ignored is that the organic fluorophores usually used in these applications suffer from photobleaching and chemical degradation, which cause an attenuation of the signal over time, requiring specific handling and special storage [7].

Recently, thermal sensing is gradually applied to LFA detection. Thermal sensing consists in the use of a heat transducer in which the heat generated is increased in the presence of the analyte, allowing detecting this thermal signal by said transducer. Polo et al. [20] explored the concept of sensing driven by plasmonic heating by the detection of cancer biomarker carcinoembryonic antigen (CEA) using a near infrared (NIR) light source to induce the heat generation using the plasmonic properties of anisotropic gold nanoparticles. Qin et al. [21] proposed a method using thermal contrast to quantify LFA using a green laser as a light source, which showed a 32-fold improvement in analytical sensitivity. In 2016, a similar thermal contrast reader [22] was developed by Wang that enhanced 8-fold analytical sensitivity in LFA quantification. The light source that is used to induce the generation of heat by the transducer can be tuned to specific wavelengths to prevent it from being affected by the presence of other molecules that do not absorb at those wavelengths, ensuring detection specificity. The use of light sources located at the NIR region of the electromagnetic spectrum allows to prevent the absorption of the light by the majority of the molecules from a biological origin, particularly blood [23]. These advantages show that plasmonic thermal sensing with NIR light source is a promising LFA detection method. However, in previous researches, no POCT device was developed employing LFA strips together with a NIR light source.

Herein, we developed a plasmonic thermal sensing-based portable device (Scheme 1a) that improves the analytical sensitivity in LFA without extra modification of the strips. The signal was amplified by putting the plasmon resonance into full play upon NIR laser irradiation. The laser wavelength in the prototype lies within the localized surface plasmon resonance (LSPR) peak of the nanoparticles (which act as light-to-heat transducers in our setting), thus generating heat in the test line. Then, the heat generation is detected by a thermal sensor located in the device which measures the heat generated by either infrared emission (radiation) or by heat conduction. The amount of heat generated is proportional to the number of nanoparticles in the test line and the irradiation power [24]. No additional operation is needed.

Thermal transfer has three main forms: conduction, convection, and radiation. In order to study the detection performance of different thermal transfer forms, we tested the conduction mode (contact) and radiation mode (non-contact) by two kinds of sensors (Scheme 1a and Additional file 1: Figure S1). The whole prototype is compact and uses embedded system technology and surface mounted components. The main factors that influence the detection ability were investigated to optimize the operating conditions. To verify the detection ability of the portable device, the LFA strips directly loaded with nanoparticles on the membrane was quantified and compared with conventional visual detection. As our detection method is temperature dependent, the effect of the ambient temperature on thermal signal detection was also studied and a calibration curve was obtained for the conduction mode. Finally, human chorionic gonadotropin (HCG) biomarkers were quantified as a model to verify the detection capabilities of thermal sensing.

## Materials and Methods

### Materials and Reagents

Phosphate-buffered saline (PBS) was purchased from Lonza®. N-(3-Dimethylaminopropyl)-N-ethylcarbodiimide hydrochloride (EDC) and heterobifunctional polyethylene glycol (HS-PEG-COOH, MW = 5000 g/mol (5 kDa)) were purchased from SIGMA®. Tween 20, Triton X100, bovine serum albumin (BSA), trehalose, poly-vinyl-pyrrolidone (PVP), *N*-hydroxysulfosuccinimide (S-NHS), sodium hydroxide, sodium chloride, gold(III) chloride hydrate, and HCG hormone were purchased from Aladdin®. Sucrose, sodium tetraborate decahydrate, boric acid, potassium iodide, and sodium thiosulfate pentahydrate were purchased from Sinopharm Chemical Reagent Co., Ltd. Sodium borohydride was purchased from Shanghai Lingfeng Chemical Reagent Co., Ltd. Anti-αHCG, anti-βHCG and anti-mouse secondary antibodies, nitrocellulose membrane (NC-a110), sample pad (glass fiber BX108), conjugation pad (glass fiber BX101), and polyvinyl chloride (PVC) surfaces were purchased from JieyYiBiotech™. 4-Morpholineethanesulfonic acid (MES) was purchased from Shanghai Majorbio. Pure ethanol was purchased from Changshu Yangyuan Chemical Co., Ltd.

### Synthesis of Nanoparticles (Gold Nanoprisms, AuNPrs)

The nanoparticles used in this study were obtained using a variation of our previously reported protocol [25] that was later improved [26]. Briefly, a volume of 220 mL of 0.5 mM Na_2_S_2_O_3_ was supplemented with 20 μL of 0.1 M KI. Then, 110 mL of the abovementioned solution was gradually added to a solution containing 2 mM HAuCl_4_ over the course of 30 s and incubated at room temperature to a total time of 4 min, moment in which the solution was supplemented with 110 mL of the remaining Na_2_S_2_O_3_+KI solution over the course of 30 s and incubated for another 4 min. Finally, 100 mL of Na_2_S_2_O_3_ without KI was added to the resulting solution and incubated for 60 min at room temperature obtaining the final prism-shaped nanoparticles. All incubation steps previously described were performed without shaking. After the synthesis, the nanoparticles were stabilized with PEG (PEGylation). The amount of PEG added to the nanoparticles was prepared in a 1:2 ratio (NPs to PEG) of the total weight of gold used in the synthesis. PEG was diluted in 1 mL Milli-Q water, and a determined volume of NaBH_4_ was then added to reach 1:1 molar ratio of PEG to NaBH_4_. The entire volume of the PEG to NaBH_4_ solution was completely added to the AuNPrs and adjusted to pH 12 with 2 M NaOH under mild mixing. Finally, the solution was sonicated for 60 min at 60 °C and then centrifuged for 15 min at 4400 G at room temperature to separate the AuNPrs from excess PEG and unreacted materials. Pellets were resuspended in Milli-Q water and centrifuged three times for 9 min at 4400 G at room temperature. These final samples were diluted to one quarter of their original volume to allow them to decant at room temperature for several weeks. After this time, the upper layer of the solution (containing a major fraction of smaller, lighter nanometric gold byproducts) could be removed from the AuNPrs which sediment at the bottom. The concentration of nanoparticles was obtained by measuring their absorbance (OD) at 400 nm by UV-Vis spectroscopy and applying a conversion factor (ε) of 11.3 mL mg^−1^ cm^−1^. This value was obtained experimentally by correlating the gold concentration obtained by ICP with the OD at 400 nm by UV-Vis of the final products of the synthesis.

### Conjugation of Nanoparticles with Anti-HCG Antibody

Briefly, 3 mL of a solution containing 0.5 mg/mL of PEGylated nanoparticles was washed three times with 0.1 M MES buffer pH 5.5 by centrifugation for 9 min at 6000 rpm in a mini-spin microfuge at room temperature. The final washed nanoparticles were resuspended in 1 mL final volume of the same buffer (0.1 M MES buffer pH 5.5), and 4 mg of EDC and S-NHS were added to the solution. The samples were then incubated for 20 min under mild mixing, centrifuged for 9 min at 6000 rpm min, and washed with MES buffer. Then, 20 μL of antibody stock (200 μg) was added to the sample and incubated 3 h at 37 °C followed by a second incubation overnight at 4 °C (no shaking). The next day, the conjugated nanoparticles were centrifuged (9 min at 6000 rpm) and washed twice with borate buffer 5 mM pH 9. Then, 25 mg of BSA was added to the solution. After 1 h incubation at room temperature under mild shaking, the sample was washed (9 min at 6000 rpm) with borate buffer supplemented with Tween 20 (5 mM pH 9) and finally stored at 4 °C until further use for no longer than 4–5 days.

After the preparation of the nanoparticles, the assembling of the test strips was carried out (described in ESI).

### Loading Nanoparticles on the Membrane of the Strips

In order to load the nanoparticles on the membrane of the strips, the concentration of the original stock of PEGylated nanoparticles (without antibody) was acquired and a series of dilutions were carried out in Milli-Q water, resulting in a range of concentrations from 0 (pure Milli-Q water without nanoparticles) up to 10 OD/mL for the maximum concentration, which corresponds to 0.9 mg/mL according to the conversion factor previously characterized by ICP-AES. For the sake of simplicity and extrapolation, the OD values were preferred over weight concentration. Thus, 2 μL of each of the aforementioned dilutions was added with a micropipette directly on the nitrocellulose membrane of the strips and left to dry at room temperature over the course ~ 2 h. The dried strips were stored at room temperature before the irradiation tests.

For the detection of the HCG antigen in the strips, a series of dilutions of the analyte (HCG) were carried out in PBS. Each strip was run by loading 5 μL of AuNPr conjugated with anti-HCG antibody into the conjugate pad, and 50 μL of the required dilution containing HCG. The strips were dried in a similar fashion as for the previous test.

### Development of the Portable Device

**Fig. 1:**
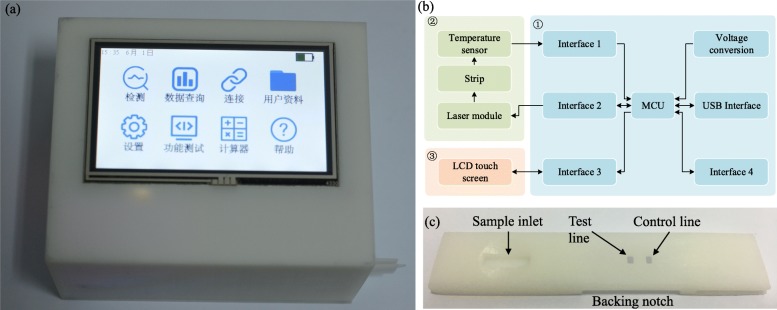
Details of portable device. **a** Plasmonic thermal sensing based portable device. **b** Hardware composition diagram ① mother board, ② laser and sensing module, and ③ user interface. **c** Cartridge for strips

The portable device (Fig. 1a and Additional file 1: Figure S1) was assembled using embedded system technology and surface mounted components, as they are of small size and cost-effective. The composition of the prototype is shown in Fig. 1b. The motherboard (Additional file 1: Figure S1) is the core module of the device, the function of which is to process the data and control the rest of the components. This module is mainly composed of the MCU STM32F407, which features a large memory and low-power operation. A voltage conversion circuit was designed on the motherboard to provide correct voltage supply for each module in the device.

Five interfaces were applied on the motherboard for the connection with other modules. The temperature sensor was connected to the motherboard through an IIC interface to receive the temperature signals transmitted by the sensor. For accurate temperature measurement, we chose temperature sensors with a digital output. The sensor for conduction mode was a semiconductor sensor (ADT7420, Analog Devices) with a 16-bit temperature resolution (0.0078 °C) and low power consumption (700 μW). In radiation mode, we made use of an infrared thermometer (MLX90614, Melexis) with a 17-bit temperature resolution and 3.9 mW power consumption. The interface between the laser control module and the motherboard consisted of a relay control circuit to ensure accurate management of the laser diode while protecting the motherboard from high current. The laser module consisted of three components: (1) the laser control component (Additional file 1: Figure S1), (2) a laser diode (Thorlabs, M9-A64-0200) which provided a light source with a wavelength of 1064 nm and an optical maximum power output of 200 mW, and (3) an aspherical lens (Thorlabs, 354330-C) mounted in the laser module to converge the light emitted by the laser diode into a 1 mm × 2.5 mm area. These components allowed to accurately illuminate the test line on the strip. An LCD touch screen (TaoJinChi Corporation TJC4827K043_01RN, 480 × 272 pixel) was utilized to provide a graphical user interface. Interface 4 of the board was left for program download and debug. A USB interface was assembled in the device, which served as a charging port for the battery and a communication port between the device and an optional external computer. The prototype was powered by a 10,000 mAh lithium battery. Programs in the MCU were compiled by IAR software (version 7.50.2.10505). The graphical user interface was designed using the USART HMI software.

### Design of the Prototype Case and Test Strip Cartridge

In order to ensure that the device is user-friendly and portable, a 3D-printed shell and a cartridge were designed, improving the anti-interference ability and stability of the device. White-colored resin was used as material for both the case and cartridge. Solidworks 2018 software was used for the design.

A cuboid case (Additional file 1: Figure S2a) and a rectangular bottom plate (Additional file 1: Figure S2b) were designed for the device according to the shape of the internal components. The cuboid casing provided a fixed mounting position for the LCD screen and the laser diode control module. A rectangular slot on the side of the housing was used for inserting the test strip cartridge. The bottom plate was provided with a battery and a motherboard mounting base, which enabled the components to be fastened to the bottom plate without moving. All detection parts were fixed in the bottom plate. A support frame for both the sensor and the strip cartridge was settled in the bottom plate, bringing them into close contact. A fine-tuning moving track was provided for the laser diode and lens allowing fixing and adjusting the distance. The size of the whole casing was 133 mm × 108 mm × 73 mm.

A special cartridge (Fig. 1c, 15 mm × 4 mm × 70 mm) was designed for the protection of test strips. The cartridge had three windows, one for the loading of the sample and two more for the visualization of the test line and control line, respectively. The window of the test line was designed slightly smaller than the width of the test strip to ensure that the laser is unable to pass through the test strip and affect the detection of the sensor. A backing notch was crafted on the back of the cartridge allowing the conductive sensor to fully touch the back of the strips in the position of the test line while ensuring that the radiation sensor could detect the temperature properly.

### Algorithm for Thermal Sensing and Parameters Calculation

Since the laser illuminated the nanoparticles, heat was generated in the test line which caused detectable temperature changes. This heat generation (*Q*, W/m^3^) depends on the concentration of nanoparticles (*C*, OD/mL), the illumination area (*A*, m^2^), and the laser intensity (*I*, W/m^2^) [22], according to the following formula:

1 $$ Q= CIA $$

The thermal signal (temperature) was collected when the laser illuminated the strips. As the illumination area and laser intensity were kept constant, the thermal signal changes with the amount of nanoparticles bind on the test line. For quantification of the heat generation, two methods were compared. The first one used the temperature changes (Additional file 1: Figure S3) to quantify the thermal signal. The variation of temperature (∆*T*) was calculated for the determination:

2 $$ \Delta  T={T}_{\mathrm{end}}-{T}_0 $$

where *T*_end_ is the final (maximum) temperature reached at the end of the irradiation and *T*_0_ is the initial ambient temperature registered by the sensor before the starting of the irradiation. Another method used the quantitative calculation of the area under the curve (AUC, Additional file 1: Figure S3). This method divides the curve into trapezoids according to a sampling frequency of 10 Hz following by the computation of the addition of all trapezoids. The thermal signal was achieved by dividing the area by the detection time (*t*_det_):

3 $$ \mathrm{AUC}=\sum \limits_{i=1}^n\left(\Delta  {T}_i+\Delta  {T}_{i-1}\right)\times 0.1\div 2 $$

4 $$ {T}_{\mathrm{auc}}= AUC\div {t}_{\mathrm{det}} $$

When applying both methods in the detection, the AUC analysis gave a better repeatability of the quantification (Additional file 1: Figure S4). Therefore, the AUC analysis was selected for its use in the final heat quantification.

To evaluate the performance of the different detection methods, we assessed the LOD of the quantification. In each experiment, we measured one concentration for four samples (four strips, *n* = 4). Considering the standard deviation (σ_0_) of the blank group and the sensitivity (*S*) which is the slope of the standard curve in linear range, we evaluated the LOD as below:

5 $$ \mathrm{LOD}=\frac{3{\sigma}_0}{s} $$

### Assay Procedure

The whole assay procedure comprised three main steps: (1) data collection, (2) detection and result acquisition, and (3) result display and storage. First, the test strip was loaded in the cartridge and inserted into the device. The measurement was carried out simply by tapping the detection button and typing the patient information (optionally, an anonymous code can be input instead). The information was transmitted to the microcontroller unit (MCU) and stored. Then, MCU activated the temperature sensor and the laser diode to begin the test. Meanwhile, the temperature data received by the MCU was sent to the LCD for real-time display and plotting. After the detection, the MCU calculated the AUC value and represented the result on the screen.

## Results and Discussion

### Characterization of the Nanoparticles

**Fig. 2:**
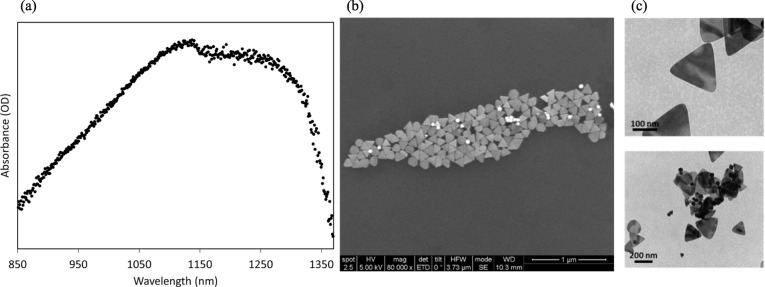
Characterization of the gold nanoprims. **a** UV-Vis spectra of the nanoparticles. Representative images of the unconjugated nanoparticles visualized by **b** SEM and **c** TEM

UV-Vis spectra of conjugated nanoprims are shown in Fig. 2a, indicating that the maximum peak is at 1130 nm. The absorbance of the AuNPr at the laser wavelength (1064 nm) is 92% percent of the maximum absorbance at 1130 nm. SEM and TEM images (Fig. 2b, c) were collected to visualize the morphology of the nanoparticles, confirming a majority of triangular shapes.

### Optimization of the Measurement Conditions in the Device

**Fig. 3:**
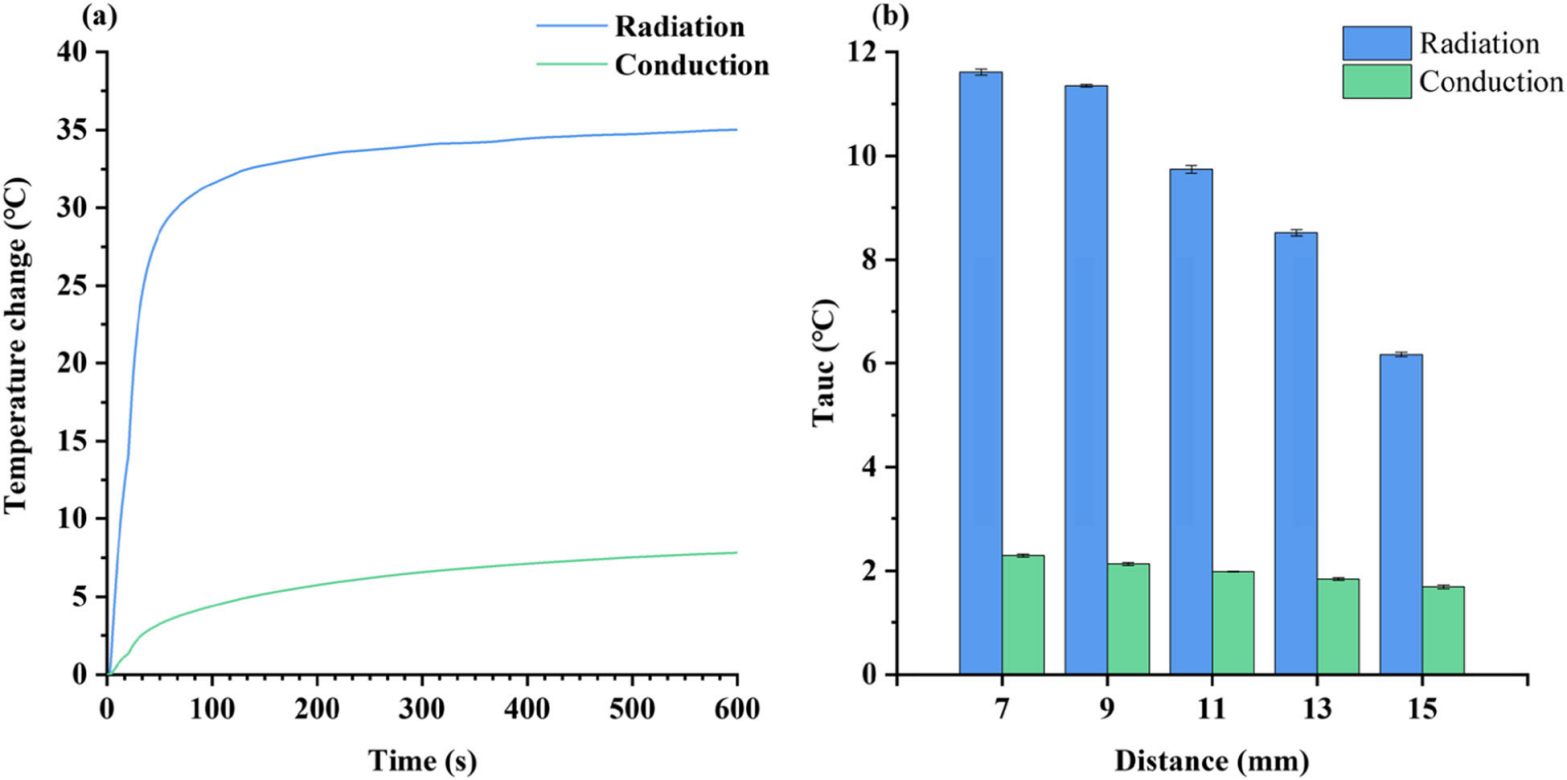
Thermal sensing optimization. **a** Temperature changes in 10 min irradiation. **b** Thermal signal at different irradiation distances

In thermal sensing, the detection time and the distance from the laser diode to the test line are the main factors that influence in the signal response [27, 28]. The two factors were studied to optimize the measurement conditions. For the optimization of the irradiation time, we irradiated the strips for 10 min and recorded the temperature changes by both sensors respectively. As can be seen from Fig. 3a, the temperature continued to rise within 10 min, but the rise in temperature started reaching a plateau after 120 s. This result matches with previous research in which a similar trend was observed in thermal signal changes with time [28]. Considering the requirements of POCT and power consumption, the detection time of the device was set to 120 s.

The optimization of the distance was then performed. Figure 3b showed that the thermal signal decreased with the increase of distance between the laser diode and the test line. The reason may be that the laser power reaching the test line was attenuated as the distance increases the effective irradiated area. In order to gain the maximum signal response, the distance was set to 7 mm.

### The Effect of Ambient Temperature on Thermal Sensing

**Fig. 4:**
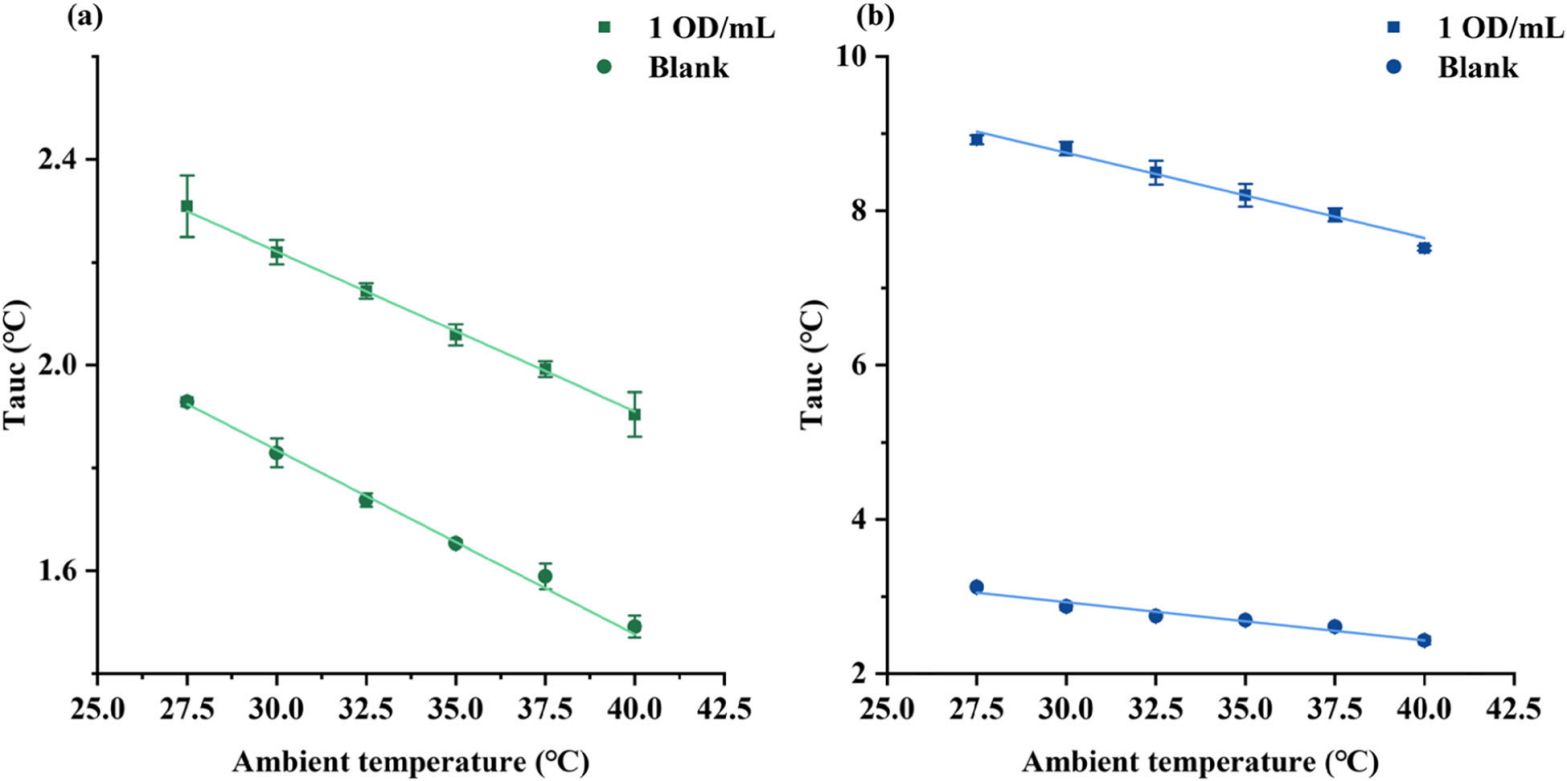
Effect of ambient temperature. **a** Thermal signal changes with ambient temperature in conduction mode. **b** Thermal signal changes with ambient temperature in radiation mode

Since thermal sensing is closely related to temperature, it was necessary to research how the ambient temperature influences the thermal sensing. The ambient temperature was ranged from 27.5 to 40 °C using an incubator. A total of 4 samples were measured at each temperature point with an interval of 2.5 °C. The ambient temperature versus thermal signal curves was measured for the blank and 1 OD/mL strips respectively by both thermal sensing methods. The fitting curve’s parameters of the ambient temperature are shown in Table 1. Figure 4a shows that in conduction mode, the curve slopes were generally consistent for different concentrations, indicating that changes in ambient temperature had the same effect on different concentrations. As a result, the ambient effect curve can be used to calibrate quantitative results. In radiation mode, the slopes of the curves (Fig. 4b) corresponding to the two concentrations were consistent with each other, but both the curves showed a downward trend. The results suggest that the conduction mode is more reliable when measuring samples under conditions with a high-temperature variability.

**Table 1:** The results of ambient temperature influence

Methods	Concentration	Fitting formula	*R*^2^
Conduction	Blank	*y* = − 0.035*x* + 2.91	0.997
	1 OD/mL	*y* = − 0.031*x* + 3.15	0.998
Radiation	Blank	*y* = − 0.050*x* + 4.42	0.962
	1 OD/mL	*y* = − 0.110*x* + 12.06	0.980

### Quantification of the Nanoparticles

#### Thermal Sensing Detection

**Fig. 5:**
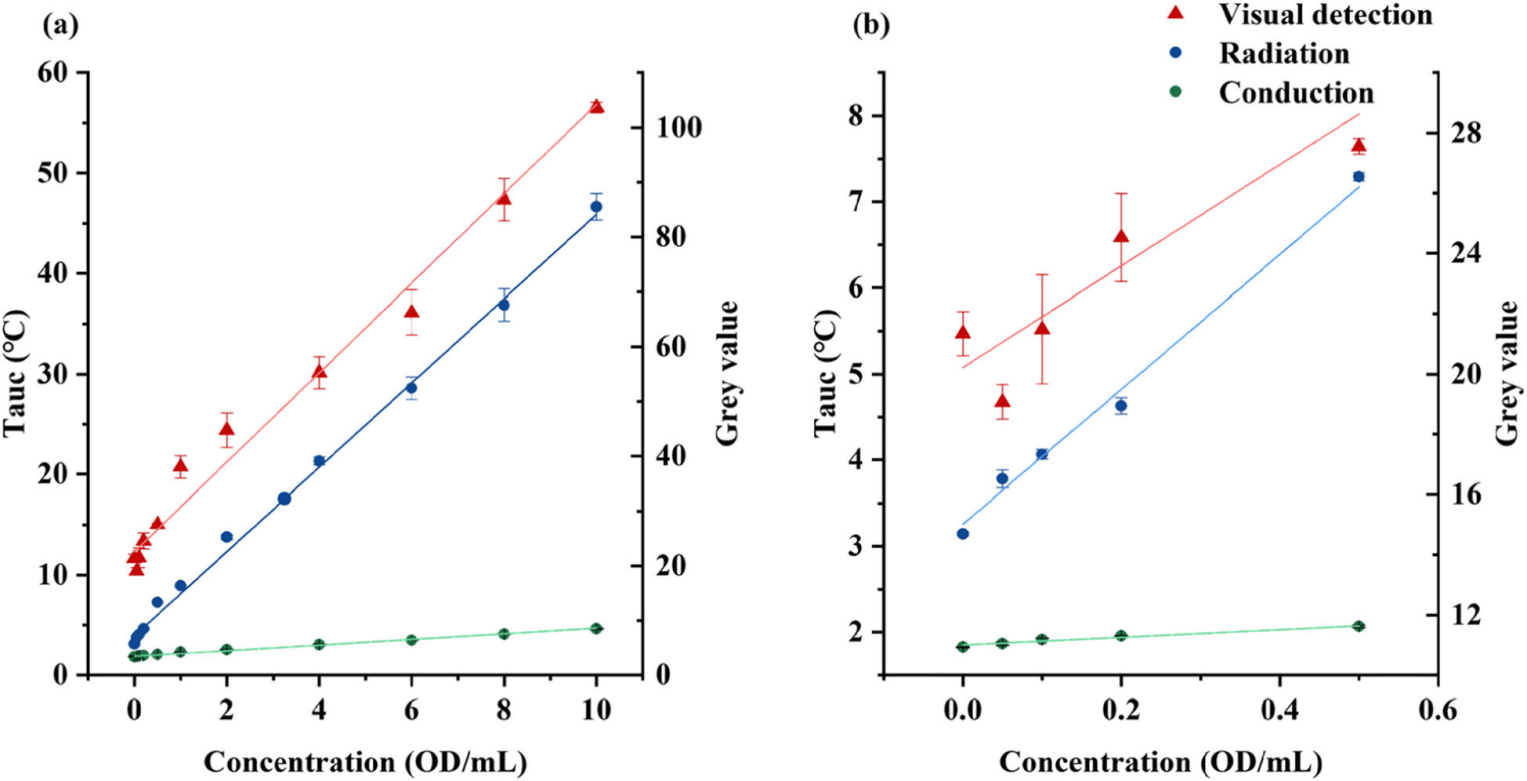
Quantification of nanoparticle. **a** Standard quantification curves in three methods. **b** Quantitative results in low concentration with linear curves

To obtain the standard quantification curves, the two thermal sensing methods (Additional file 1: Figure S5) were used respectively to detect test strips containing nanoparticles at the range from 0 to 10 OD/mL. Those strips (Additional file 1: Figure S6a) containing different concentrations of nanoparticles were detected by the portable device mentioned (see the “Materials and Methods” section). The setting of both sensors in the device is presented in Additional file 1: Figure S5a and S5b. Four samples were tested for each concentration at an ambient temperature of 27.5 °C. The device applied the AUC method to calculate the thermal signal at the test line. Therefore, the quantification of the thermal signal was proportional to the amount of nanoparticles on the test line. The quantification curves (Fig. 5a) were generated by linear regression of the data obtained from the thermal signal against the concentration of the nanoparticles and are represented by the formulae in Table 2.

**Table 2:** The results of different methods in nanoparticle quantification

Methods	Fitting formula	*R*^2^	Sensitivity	LOD
Conduction	*y* = 0.28*x* + 1.87	0.981	0.28	0.053
Radiation	*y* = 4.20*x* + 3.93	0.997	4.20	0.023
Visual detection	*y* = 8.17*x* + 22.57	0.988	8.17	0.268

Table 2 shows that the sensitivity of the radiation mode was 15-fold higher than that of the conduction mode. The LOD of conduction and radiation modes were 0.053 OD/mL and 0.023 OD/mL respectively. In thermal sensing, the radiation mode improved the limit of detection (LOD) by 2-fold compared with conduction mode. The stability of LFA strips was also tested as depicted in Additional file 1: Figure S7. The conductive thermal sensing mode requires the transfer of heat between two solids. When the temperature of the test line raised rapidly, it takes time (relaxation time) for the sensor to reach the same temperature as the test line. As a result, the temperature of the sensor was lower than the actual temperature in the test line at the end of detection. On the other hand, the radiation mode did not require a transfer of the heat to the sensor, as the sensor directly detected the IR wave irradiated by the test line to obtain its current temperature. In the conduction mode of detection, part of the heat was dissipated due to the presence of good thermal conductors which worked as a heatsink, while in the radiation detection, only air and the strip itself intervened in the heat dissipation. These reasons can explain that the sensitivity of the conduction method is lower than the radiation method.

When testing strips with a concentration of 10 OD/mL, we found that there was a burning mark using the radiation (non-contact) sensing mode. One possible reason for this phenomenon is that in the non-contact measurement, the low thermal conductivity of the air allows the heat to be retained in the test line and dissipate less efficiently, increasing the effective local temperature and eventually causing the combustion of the membrane.

In the contact mode of detection, however, the sensor with a large thermal conductivity acted as medium and heatsink. In this way, heat was conducted to the sensor so that no combustion occurred in the test line.

#### Comparison Between Thermal Sensing and Visual Detection

Due to its popularity for portable devices and wide use, we compared the thermal sensing with visual detection for its detection ability. For visual detection, the pictures of the strip were taken by a conventional microscope digital camera. The test strips were mounted in the cartridge to ensure the positional consistency of the image analysis in a similar fashion than with the thermal sensing. Software Image J was used to analyze the grey value in the test line for different concentrations of nanoparticles. A standard curve (Fig. 5a) of the visual detection method was plotted based on the results of this analysis. The linear range between the grey value and the concentration of nanoparticles was 0.2–10 OD/mL (*R*^2^ was 0.770 for the range of 0–0.2 OD/mL, so they were thus discarded from further analysis). The detection limit was 0.268 OD/mL. The results indicated that thermal sensing could reduce LOD by 5- to 12-fold compared to visual detection. In Qin’s research, they found that the LOD for visual analysis was 100-fold higher than thermal contrast [21]. Since they employed a high laser power and an infrared camera, they gained greater difference in LOD. One reasonable explanation for the LOD improvement is that thermal sensing is able to measure the nanoparticles on top and beneath the membrane surface. Another advantage of thermal sensing is that it has a higher stability than visual detection. Thermal sensing generates heat by the nanoparticles on the entire test line. Visual detection relies only on the color reaction of the nanoparticles on the surface of the test line. Even if the analyte concentrations of two test strips are the same, the distribution of the nanoparticles on the T-line in the tangent plane is different; thus, the visual inspection will result in a difference in the detection results while the thermal sensing is more stable and reproducible. On the contrary, the sensitivity of the visual detection was 2-fold higher than thermal sensing. Visual detection is a direct method for quantifying nanoparticles, while thermal sensing is an indirect measurement of the concentration of the nanoparticles by measuring the temperature changes, which may partially explain the lack of sensitivity. Figure 5b demonstrates that the linear range of detection for thermal sensing can be as low as 0 OD/mL, with the *R*^2^ of 0.972 (conduction) and 0.987 (radiation), suggesting that thermal sensing has a better potential for its applications in early detection in POCT than color quantification, since the target analytes are in lower concentrations.

### Quantitative Detection of HCG

**Fig. 6:**
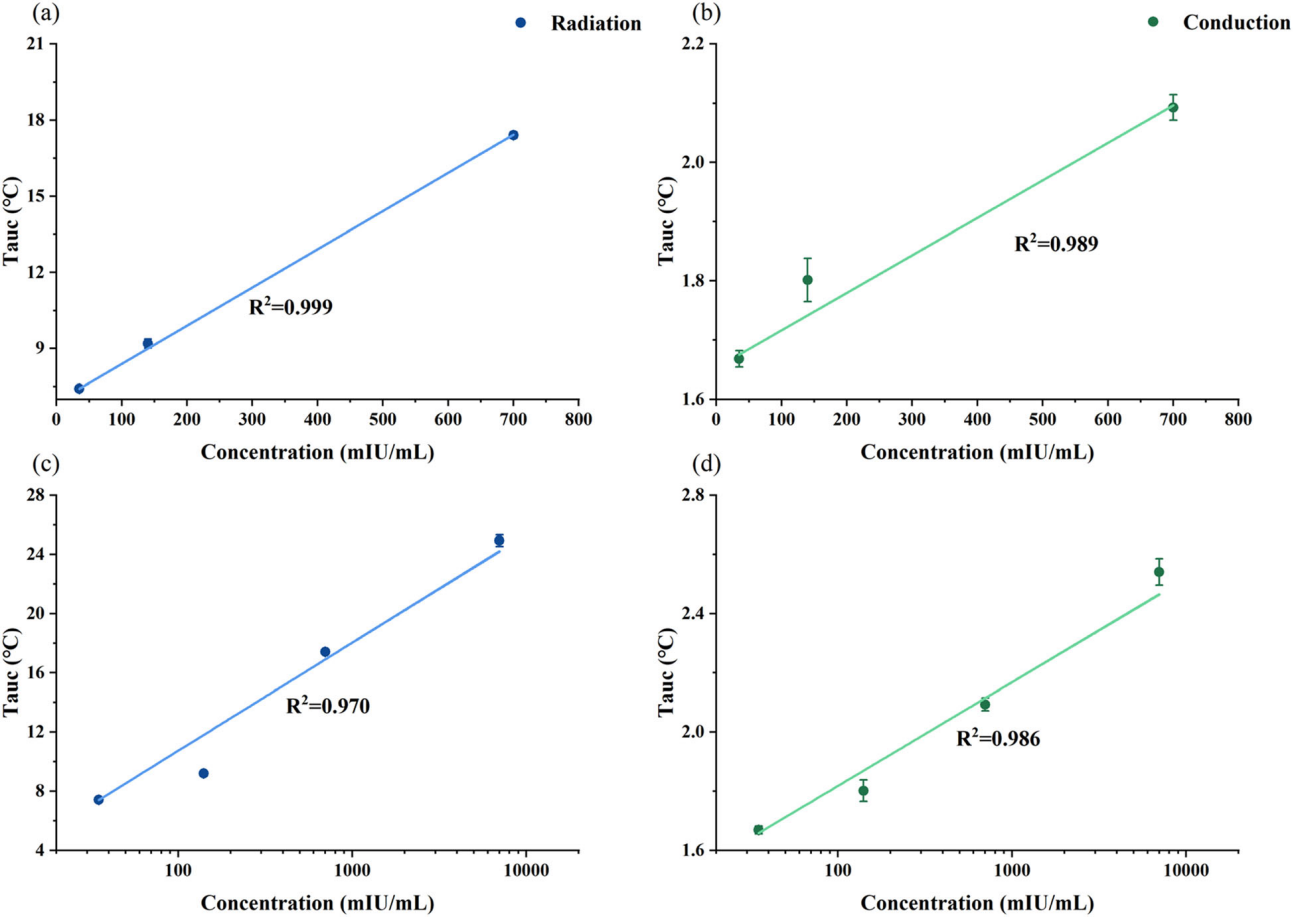
The standard curves of HCG. **a** A linear curve between the logarithm of the HCG concentration and the thermal signal in radiation mode. **b** A linear curve between the logarithm of the HCG concentration and the thermal signal conduction mode. **c** The quantification results of HCG in radiation mode. **d** The quantification results of HCG in conduction mode

Finally, the biomarker HCG was quantified using our system in order to validate the thermal sensing. Both conduction and radiation modes were applied to quantify the HCG. The optical power was turned down to 150 mW, preventing the strips from burning. Strips (Additional file 1: Figure S6b) with four different concentrations were tested. Figure 6a and b show that the thermal signals were linear to the concentration of HCG from 35 to 700 mUI/mL. When the concentration was extended to the range of 35–7000 mUI/mL, the linearity was between the logarithm of the concentration and the thermal signal as in Fig. 6c, d. In conduction mode, the LOD was 64.2 mIU/mL which is in a similar range than the visual detection. However, the ideal LOD of the radiation mode was 2.8 mIU/mL. The data matched with the quantification of nanoparticles. Compared with other devices that applied photothermal effect (LOD = 5.5 mIU/mL) [27], our device in radiation mode reduced the LOD by nearly 2-fold. Those results proved that thermal sensing is an effective way in LFA detection and quantification.

## Conclusions

A plasmonic thermal sensing method for LFA detection was established. A portable device based on this method was developed by applying different temperature sensors (conduction and radiation modes). The study of the influence of the ambient temperature demonstrated that it has a negative impact on the thermal sensing and conduction mode was less affected than radiation mode. In radiation mode, the impact was more significant at high concentrations. Both modes were also tested to compare the quantification ability. When compared with the traditional visual detection, the thermal sensing methods showed a 5- to 12-fold improvement in LOD for nanoparticle quantification. The radiation mode showed a better performance than conduction mode in both sensitivity and LOD. In the validation of thermal sensing, LFA strips for the detection of HCG were tested and the results demonstrated that the radiation mode was much more sensitive than the conduction mode. In this way, we proved that thermal sensing is a feasible and effective way for early detection in LFA platforms.

In conclusion, plasmonic thermal sensing can truly improve the analytical sensitivity and shows a promising future in LFA detection for early diagnostic applications. The portable device described herein provided two sensing approaches to satisfy different requirements.

## Additional File

**Additional file 1: Figure S1.** Internal structure and components in the device ①motherboard ②sensor ③laser control component ④laser diode ⑤aspherical lens ⑥LCD touch screen ⑦lithium battery (inside). **Figure S2.** 3D model of the case (a) Bottom view of the case (b) 3D side view of the bottom plate. **Figure S3.** Two algorithm methods for thermal signal. Green arrow line segment represents temperature change algorithm. The blue area reperenets the area under the curve (Tauc). **Figure S4.** Thermal signal calculated by two methods of 0 OD/mL. The labels in blue represent the results measured under radiation forms. The labels in green represneted the results measured under conduction form. The figure represents the results of 0 OD/ml for 4 times test. The results variation of the measurement by *T*_*auc*_ method A was smaller than the *∆T* method, showing that *T*_*auc*_ methods had better repeatablity. And the *T*_*auc*_ is little influenced by the noise since the thermal signal of 0 OD/ml is lower than *∆T*. **Figure S5.** The setting of two different sensors (a) Sensor setting in conduction form (b) Sensor setting in radiation form. **Figure S6.** Strips used in quantification. (a) Strips merely containing nanoparticles. The concentration of nanoparticles strips from A to control was: 10 OD/mL, 8 OD/mL, 6 OD/mL, 4 OD/mL, 2 OD/mL, 1 OD/mL, 0.5 OD/mL, 0.2 OD/mL, 0.1 OD/mL, 0.05 OD/mL, 0 OD/mL (control), respectively. (b) Strips with HCG. The concentration of HCG for 200K to 1K was: 35 mIU/mL, 140 mIU/mL, 700 mIU/mL, 7000 mIU/mL, respectively. **Figure S7.** Stability of LFA strips (a) Test in conduction mode (b) Test in radiation mode. The strips with 4 OD/mL and 1 OD/mL were used in the stability experiment. Each concentration has 4 strips and was divided into 4 groups (2 concentrations for each of the two modes). Each strip was tested for 5 times and the standard deviations were caculated.

## Data Availability

The datasets used and/or analyzed during the current study are available from the corresponding author on reasonable request.

## Abbreviations

AUC: Area under the curve
AuNPrs: Gold nanoprisms
BSA: Bovine serum albumin
CCD: Charge-coupled device
CMOS: Complementary metal oxide semiconductor
HCG: Human chorionic gonadotropin
LFA: Lateral flow assay
LOD: Limit of detection
LSPR: Localized surface plasmon resonance
MCU: Microcontroller unit
MES: 4-Morpholineethanesulfonic acid
NIR: Near infrared
PBS: Phosphate-buffered saline
POCT: Point-of-care testing
PVC: Polyvinyl chloride
PVP: Poly-vinyl-pyrrolidone
S-NHS: *N*-Hydroxysulfosuccinimide
