# Supplementary material for: A plasmonic thermal sensing based portable device for lateral flow assay detection and quantification
Source: Nanoscale Res Lett. 2020 Jan 13;15:10. doi: 10.1186/s11671-019-3240-3 (PMC6957652; doi:10.1186/s11671-019-3240-3)
Supplement: Supplementary file 1 — Additional file 1: Figure S1. Internal structure and components in the device ①motherboard ②sensor ③laser control component ④laser diode ⑤aspherical lens ⑥LCD touch screen ⑦lithium battery (inside). Figure S2. 3D model of the case (a) Bottom view of the case (b) 3D side view of the bottom plate. Figure S3. Two algorithm methods for thermal signal. Green arrow line segment represents temperature change algorithm. The blue area reperenets the area under the curve (Tauc). Figure S4. Thermal signal calculated by two methods of 0 OD/mL. The labels in blue represent the results measured under radiation forms. The labels in green represneted the results measured under conduction form. The figure represents the results of 0 OD/ml for 4 times test. The results variation of the measurement by Tauc method A was smaller than the ∆T method, showing that Tauc methods had better repeatablity. And the Tauc is little influenced by the noise since the thermal signal of 0 OD/ml is lower than ∆T. Figure S5. The setting of two different sensors (a) Sensor setting in conduction form (b) Sensor setting in radiation form. Figure S6. Strips used in quantification. (a) Strips merely containing nanoparticles. The concentration of nanoparticles strips from A to control was: 10 OD/mL, 8 OD/mL, 6 OD/mL, 4 OD/mL, 2 OD/mL, 1 OD/mL, 0.5 OD/mL, 0.2 OD/mL, 0.1 OD/mL, 0.05 OD/mL, 0 OD/mL (control), respectively. (b) Strips with HCG. The concentration of HCG for 200K to 1K was: 35 mIU/mL, 140 mIU/mL, 700 mIU/mL, 7000 mIU/mL, respectively. Figure S7. Stability of LFA strips (a) Test in conduction mode (b) Test in radiation mode. The strips with 4 OD/mL and 1 OD/mL were used in the stability experiment. Each concentration has 4 strips and was divided into 4 groups (2 concentrations for each of the two modes). Each strip was tested for 5 times and the standard deviations were caculated. [file 11671_2019_3240_MOESM1_ESM.docx]

Electronic Supplementary Information

A plasmonic thermal sensing based portable device for lateral flow assay detection and quantification

Zhuo Qu ^1^, Kan Wang ^1^,Gabriel Alfranca^1,3*^, Jesús M. de la Fuente^1,2,3*^, Daxiang Cui ^1*^

*Correspondence: dxcui@sjtu.edu.cn, jmfuente@unizar.es, alfrancagabriel@gmail.com

1、Institute of Nano Biomedicine and Engineering, Shanghai Engineering Research Center for Intelligent diagnosis and treatment instrument, Department of Instrument Science and Engineering, School of Electronic Information and Electrical Engineering, Shanghai Jiao Tong University, 800 Dongchuan Rd, Shanghai 200240, China.

2、Instituto de Ciencia de Materiales de Aragón (ICMA), CSIC/Universidad de Zaragoza, C/Pedro Cerbuna 12, 50009 Zaragoza, Spain.

3、Centro de Investigación Biomédica en Red de Bioingeniería, Biomateriales Nanomedicina (CIBER-BBN), 50018 Madrid, Spain.

### Assembling of the test strips

All the equipment used during the construction of the strips was thoroughly washed and rinsed with ethanol prior use. All the manipulation of the pads, PVC and NC was performed using tweezers and gloves and avoiding direct contact with the strip (as far as possible).

Briefly, the sample and conjugate pads were cut (30 cm ×1 cm for the conjugate pad and 30 cm×2.5 cm for the sample pad) and incubated at room temperature, first with Milli-Q water for 15 min, and then with pure ethanol for 15 min. Then both pads were dried at 37 °C for 1 hour, incubated with their respective buffers at room temperature for 1 hour and finally dried overnight at 37 °C. The buffers applied to each pad consisted of 20 mM borate buffer pH 7.4, 2% NaCl, 5% BSA, 0.2% PVP and 0.05% Triton X100 for the sample pad; and 20 mM borate buffer pH 7.4, 5% Sucrose, 2% Trehalose and 0.05% Triton X100 for the conjugate pad.

Meanwhile, the NC membrane (30 cm × 2 cm) and the absorbent pad (30 cm × 3 cm) were attached to the middle and upper area of a 30 x 8 cm PVC surface, respectively. Note that the absorbent pad must overlap 2-3 mm the NC membrane. Then, the antibodies were loaded to the NC using a Biodot automatic dispenser, forming two straight lines of each antibody, corresponding to the TL (2 mg/mL anti-βHCG) and CL (1.5 mg/mL anti-mouse antibody) both previously supplemented with 50% sucrose (2% final concentration). The resulting structure was dried at 37 °C for 2 hours. After this, the conjugate and sample pads were attached to the lower part of the PVC surface, overlapping 2-3 mm with each other and with the NC membrane. Finally, the whole structure was cut using an automatic cutter obtaining 0.3 cm strips. The resulting LFA strips were stored at room temperature in a dry container with silica beads.

### Details in the portable device


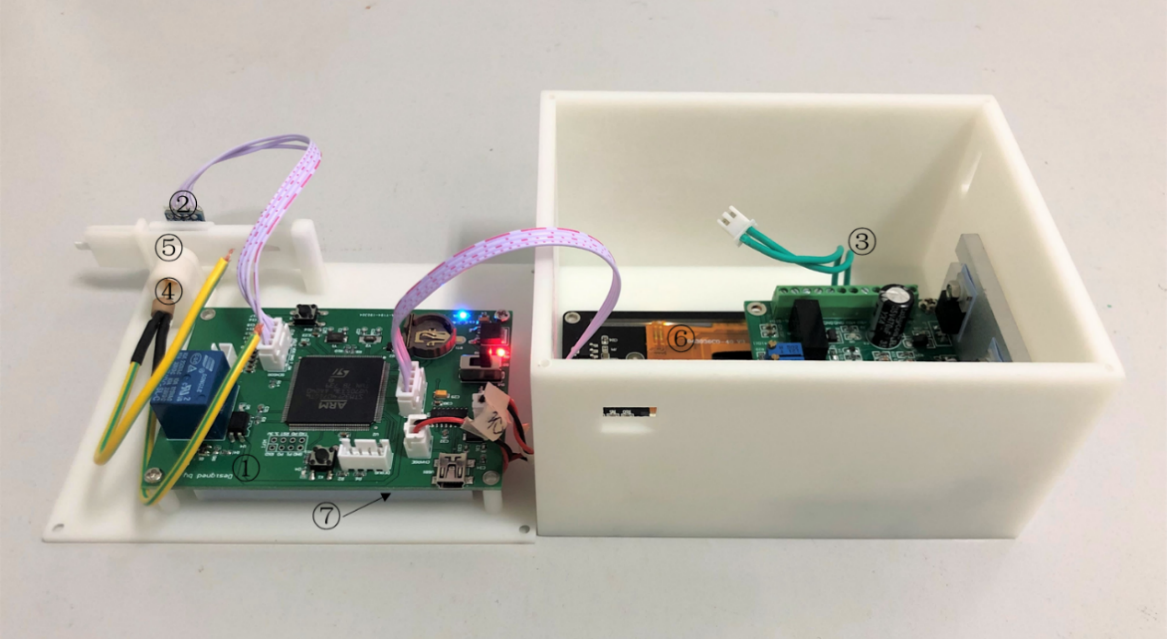


Figure S1 Internal structure and components in the device ①motherboard ②sensor ③laser control component ④laser diode ⑤aspherical lens ⑥LCD touch screen ⑦lithium battery (inside)


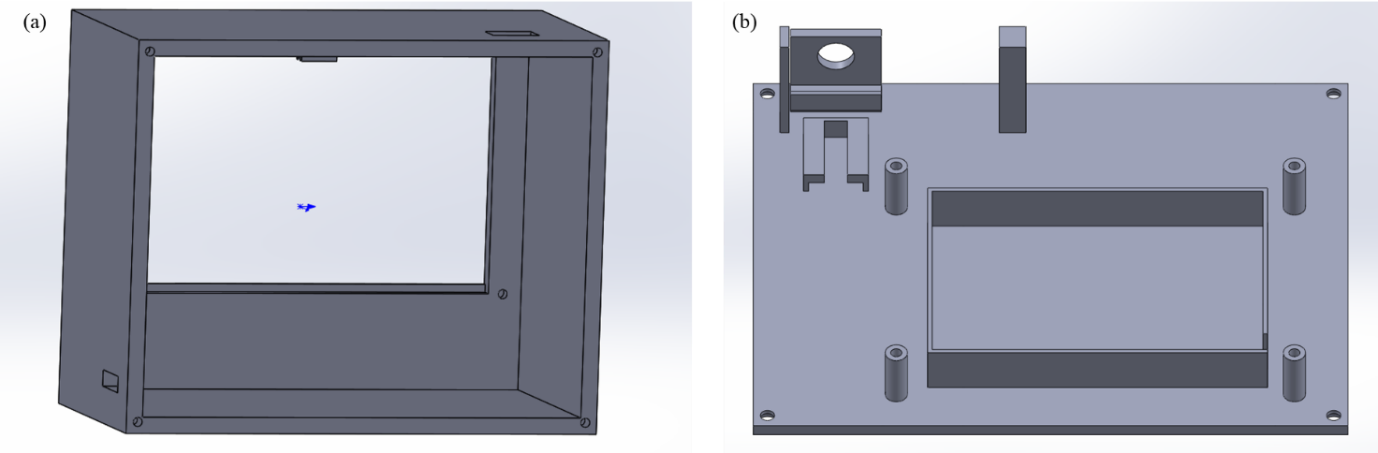


**Figure S2** 3D model of the case (a) Bottom view of the case (b) 3D side view of the bottom plate


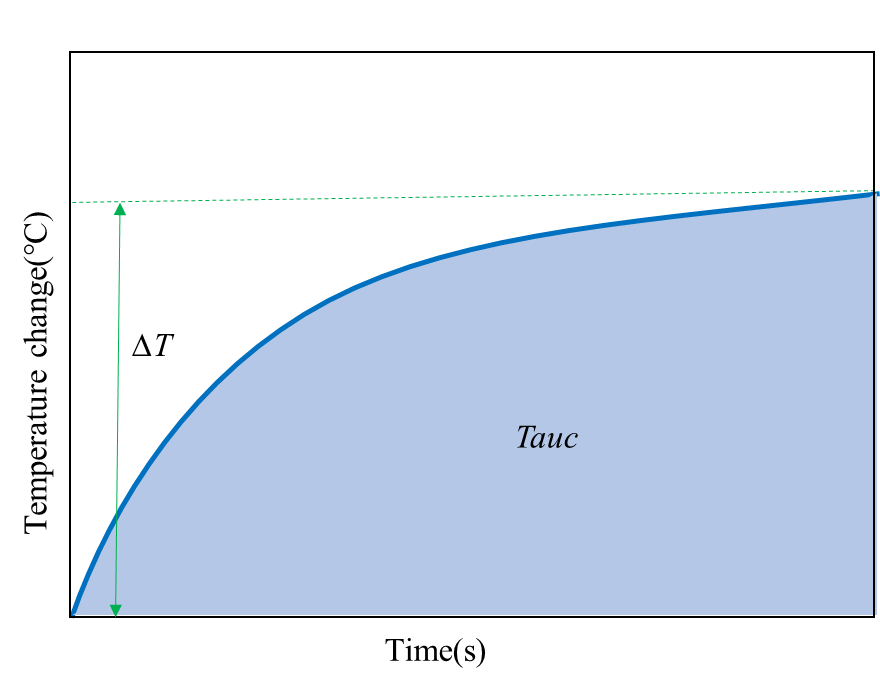


Figure S3 Two algorithm methods for thermal signal. Green arrow line segment represents temperature change algorithm. The blue area reperenets the area under the curve (Tauc).





Figure S4 Thermal signal calculated by two methods of 0 OD/mL. The labels in blue represent the results measured under radiation forms. The labels in green represneted the results measured under conduction form.

The figure represents the results of 0 OD/ml for 4 times test. The results variation of the measurement by $T_{auc}$ method A was smaller than the $\Delta T$ method, showing that $T_{auc}$ methods had better repeatablity. And the $T_{auc}$is little influenced by the noise since the thermal signal of 0 OD/ml is lower than $\Delta T$


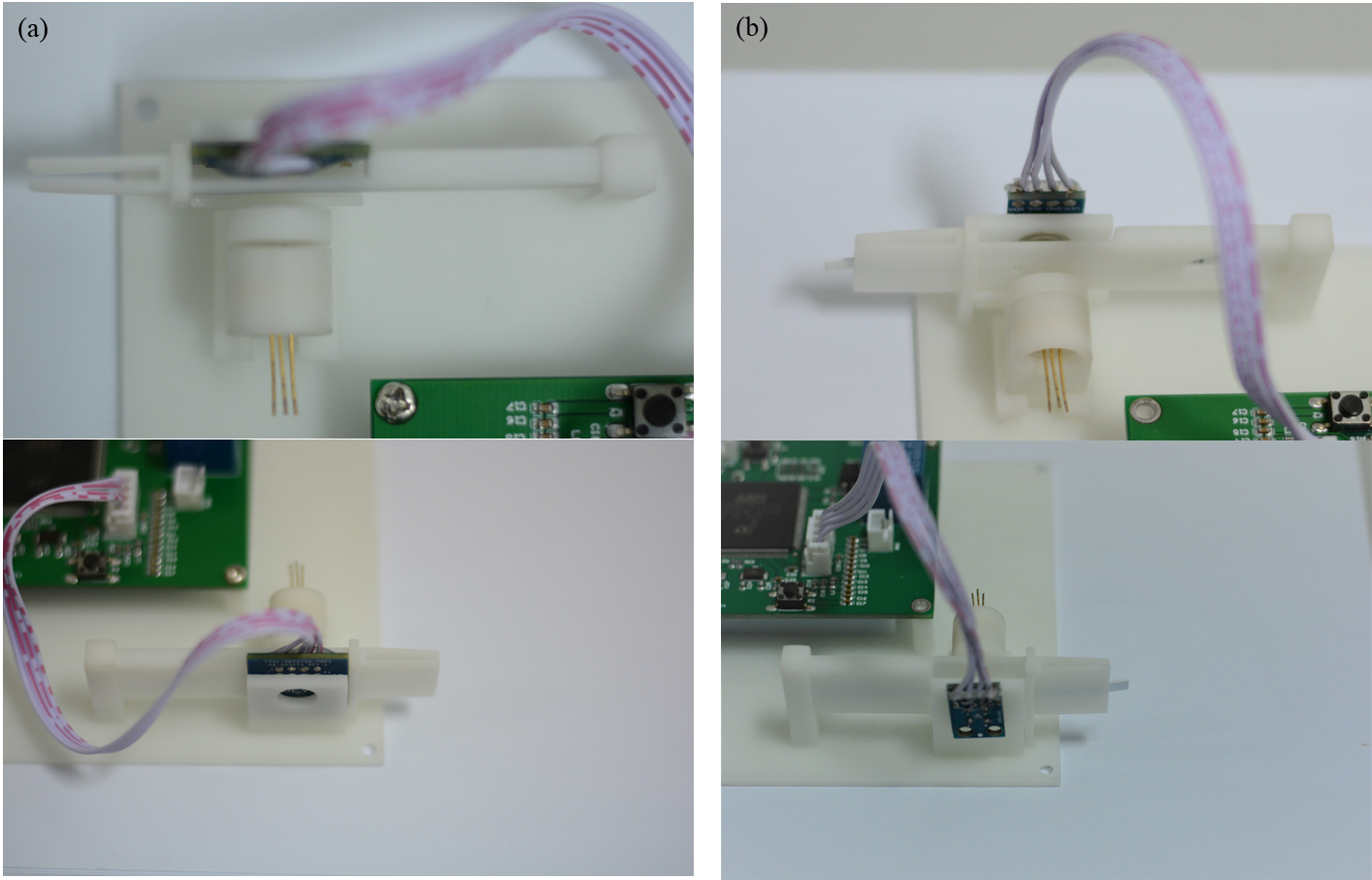


**Figure S5** The setting of two different sensors (a) Sensor setting in conduction form (b) Sensor setting in radiation form


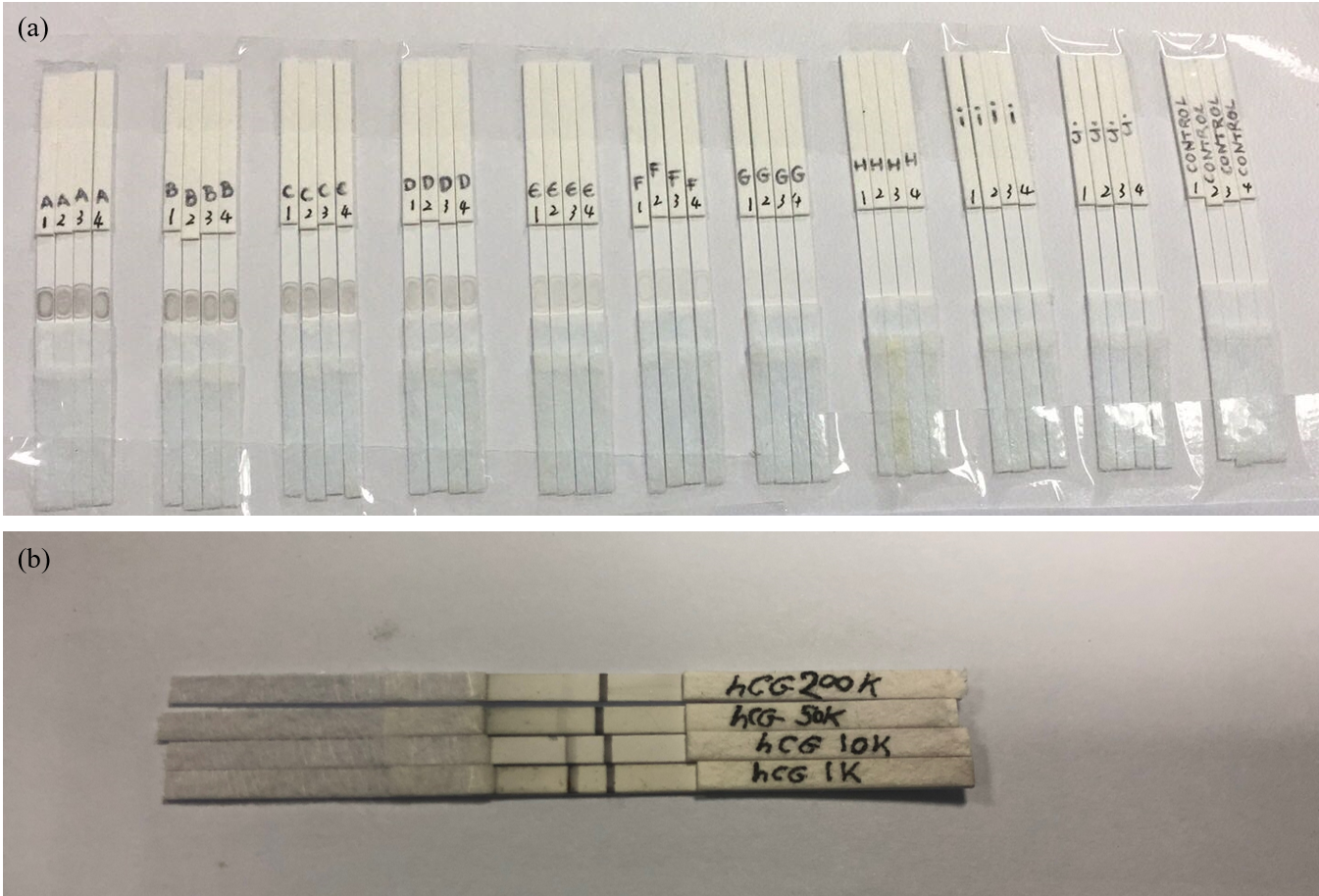


**Figure S6** Strips used in quantification. (a) Strips merely containing nanoparticles. The concentration of nanoparticles strips from A to control was: 10 OD/mL, 8 OD/mL, 6 OD/mL, 4 OD/mL, 2 OD/mL, 1 OD/mL, 0.5 OD/mL, 0.2 OD/mL, 0.1 OD/mL, 0.05 OD/mL, 0 OD/mL (control), respectively. (b) Strips with HCG. The concentration of HCG for 200K to 1K was: 35 mIU/mL, 140 mIU/mL, 700 mIU/mL, 7000 mIU/mL, respectively.


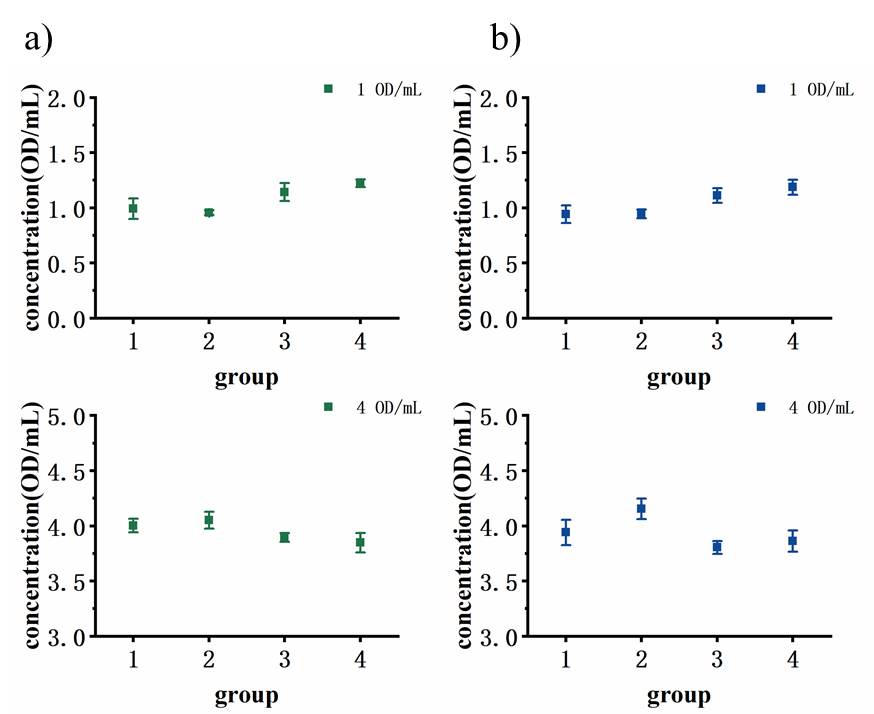


**Figure S7** Stability of LFA strips a) Test in conduction mode b) Test in radiation mode. The strips with 4 OD/mL and 1 OD/mL were used in the stability experiment. Each concentration has 4 strips and was divided into 4 groups (2 concentrations for each of the two modes). Each strip was tested for 5 times and the standard deviations were caculated.
